# Korea hypertension fact sheet 2021: analysis of nationwide population-based data with special focus on hypertension in women

**DOI:** 10.1186/s40885-021-00188-w

**Published:** 2022-01-03

**Authors:** Hyeon Chang Kim, Hokyou Lee, Hyeok-Hee Lee, Eunsun Seo, Eunji Kim, Jiyen Han, Ja-Young Kwon, for the Korean Society of Hypertension (KSH)–Hypertension Epidemiology Research Working Group, Hyeon Chang Kim, Song Vogue Ahn, Sun Ha Jee, Sungha Park, Hae-Young Lee, Min Ho Shin, Sang-Hyun Ihm, Seung Won Lee, Hokyou Lee, Jong Ku Park, Il Suh, Tae-Yong Lee

**Affiliations:** 1 Department of Preventive Medicine, Yonsei University College of Medicine, Seoul, Republic of Korea; 2 Department of Internal Medicine, Yonsei University College of Medicine, Seoul, Republic of Korea; 3 Department of Public Health, Yonsei University Graduate School, Seoul, Republic of Korea; 4 Department Obstetrics and Gynecology, Yonsei University College of Medicine, Seoul, Republic of Korea

**Keywords:** Hypertension, Prevalence, Awareness, Therapeutics, Antihypertensive agents, Women, Korea

## Abstract

**Background:**

The Korean Society of Hypertension has published the Korea Hypertension Fact Sheet 2021 to provide an overview of the magnitude and management status of hypertension and their recent trends.

**Methods:**

The Fact Sheets were based on the analyses of Korean adults aged 20 years or older of the 1998–2019 Korea National Health and Nutrition Examination Survey and the 2002–2019 National Health Insurance Big Data.

**Results:**

Currently, the population average of systolic/diastolic blood pressure was 119/76 mmHg in Korean adults aged 20 years or older showing little change in the recent decade. It is estimated that 28% of the adult population aged 20 or older (33% of adults aged 30 or older) have hypertension. The estimated number of people with hypertension was 6.30 million for men and 5.77 million for women, and 1.96 million for men and 2.99 million for women among the population aged 65 or older. The number of people diagnosed with hypertension increased from 3.0 million in 2002 to 10.1 million in 2019. During the same period, the number of people using antihypertensive medication increased from 2.5 million to 9.5 million, and the number of people adherent to treatment increased from 0.6 million to 6.9 million. Among antihypertensive prescriptions, 40.6% of the patients received monotherapy, 43.4% received dual therapy, and 16.0% received triple or more therapy. The most commonly prescribed antihypertensive medication was angiotensin receptor blockers (ARB), followed by calcium channel blockers (CCB) and diuretics. In young women, angiotensin-converting enzyme inhibitors (ACEi), ARB and CCB are less frequently prescribed than in men, but 59.5% of hypertensive women aged 20–39 are prescribed ACEi or ARBs. Hypertensive disorders during pregnancy have been increasing over the past 10 years. In 2019, 5.4% of women who gave birth were diagnosed with chronic hypertension and 3.1% with pregnancy-induced hypertension.

**Conclusions:**

To achieve further improvement in management of hypertension, we need to encourage awareness and treatment in young adults. It is required to develop tailored prevention and management strategies that are appropriate for and inclusive of various demographics.

**Supplementary Information:**

The online version contains supplementary material available at 10.1186/s40885-021-00188-w.

## Background

Hypertension is the most important modifiable risk factor yet also the biggest contributor to cardiovascular and cerebrovascular diseases [1, 2]. In the twenty-first century, cardiovascular and cerebrovascular diseases mortality accounts for nearly a half of all deaths in the developed regions and for one quarter in the developing regions [3]. The cardiovascular disease mortality rate has been decreasing in Korea; nonetheless, heart disease, cerebrovascular disease and hypertensive disorders remain as the second, fourth and ninth common causes of death, respectively [4]. Moreover, due to the rapid aging of the population and improved prognosis from cardiovascular event, the absolute number of patients with cardiovascular disease continues to increase [5]. According to the National Health Insurance Service (NHIS) statistics in Korea, the estimated medical cost of treating hypertension was 3830 billion Korean Won, accounting for 4% of all medical expenses or 16% of medical expenses for chronic diseases [6]. Hitherto, controlling blood pressure is crucial not only to reduce the burden of disease at a societal level but also to improve the quality of life at an individual level. Continuous monitoring of hypertension prevalence and management status should be the first step in reducing its burden. To achieve this, the Korean Society of Hypertension had published its first Hypertension Fact Sheet in 2018, and have been periodically updating it thereafter [7, 8].

## Methods

The Korea Hypertension Fact Sheet 2021 analyzed two nationally representative datasets. The first one is the Korea National Health and Nutrition Examination Survey (KNHANES) from 1998 to 2019. The KNHANES is a national surveillance system in Korea that assesses the health and nutritional status of noninstitutionalized Korean population since 1998 [9]. The second one is the National Health Insurance (NHI) Big Data from 2002 to 2018. Organized by the NHIS, the NHI Big Data contains socio-demographics, hospital claims with International Classification of Diseases, 10th Revision (ICD-10, I10) coding, and mortality data of the entire population of Republic of Korea [8]. Previously, the Korea Hypertension Fact Sheet 2018 analyzed adults aged 30 years from the KNHANES data and people of all age in the NHI Big Data. Since the Korea Hypertension Fact Sheet 2020, both NHANES and NH-BD were analyzed for adult data aged 20 or older [10].

The English version of the “Korea Hypertension Fact Sheet 2021” is attached as a supplementary material of this manuscript. The Korean version is available at http://www.koreanhypertension.org/reference/guide.

### Analysis of the KNHANES from 1998 to 2018

There have been 8 rounds of KNHANES between 1998 and 2018: KNHANES I (1998), KNHANES II (2001), KNHANES III (2005), KNHANES-IV (2007–2009), KNHANES V (2010–2012), KNHANES VI (2013–2015), KNHANES VII (2016–2018), and KNHANES VIII (2019–2021). However, only 2019 data are currently available for the 8th round. Hypertension was defined as systolic blood pressure (SBP) ≥140 mmHg, diastolic blood pressure (DBP) ≥90 mmHg [11], or self-reported use of antihypertensive medication for the purpose of blood pressure control. Awareness rate was defined as the proportion of people with physician diagnosis of hypertension among all people with hypertension. Treatment rate was defined as the proportion of people using antihypertensive drugs for 20 days or more per month among all people with hypertension. Control rate was defined as the proportion of people with SBP < 140 mmHg and DBP < 90 mmHg among 1) all people with hypertension and 2) people treated for hypertension [9, 12]. To evaluate the magnitude and management status of hypertension without the effects of population aging, age-standardized rates were calculated based on the demographics of the Korean population in 2005 according to the Population and Housing Census, Statistics Korea. To take into account the effect on estimator variance attributable to the KNHANES’ stratified multistage clustered probability sampling design, we applied survey sampling weights to all the analyses.

### Analysis of the NHI big data from 2002 to 2018

A repeated cross-sectional analysis of 124 million cases of hypertension (assuming 1 case per calendar year if observed more than once) was conducted. While the KNHANES data analysis defined hypertension based on measured blood pressure levels and use of antihypertensive medication, the NHI Big Data analysis defined hypertension based on diagnosis codes, because the claim database did not have records of blood pressure measurements. Healthcare utilization was defined as at least one health insurance claim for diagnosis of essential hypertension (I10) each year. Treatment of hypertension was defined as at least one health insurance claim for hypertension diagnosis with antihypertensive drug prescription each year. Adherence to treatment was defined as receiving prescriptions of antihypertensive drugs ≥290 days (80%) each year. Antihypertensive drugs were classified into diuretics (thiazide-related and loop diuretics), beta-blockers, calcium channel blockers (CCB), angiotensin-converting enzyme inhibitors (ACEi), angiotensin receptor blockers (ARB), potassium-sparing diuretics, or others (alpha-blockers, vasodilators, etc.). If the regimen of antihypertensive drug had switched in a year, one with the longest duration was selected as the representative prescription of the patient for the given year. Complication screening rates were calculated for blood test (≥1 serum creatinine test) and ≥ 1 routine urinalysis and/or urine microalbumin test each year, separately. For the analyses of hypertensive disorders of pregnancy, childbearing women of age 15–49 years were extracted based on delivery procedure codes. Diagnoses of chronic hypertension, pregnancy-induced hypertension, unspecified hypertension, and preeclampsia/eclampsia were assigned to each delivery case according to the operational definitions in Table 1.

**Table 1:** Definitions of hypertensive disorders of pregnancy

Diagnosis	Definition
Chronic hypertension (A)	Having insurance claims for diagnosis of (1) hypertension (ICD-10: I10-I15) before delivery, or (2) chronic maternal hypertension (ICD-10: O10) or preeclampsia superimposed on chronic hypertension (ICD-10: O11) before or within 12 weeks after delivery
Pregnancy-induced hypertension (B)	Not meeting criteria for chronic hypertension; AND Having insurance claims for diagnosis of gestational hypertension (ICD-10: O13) or preeclampsia/eclampsia (ICD-10: O14, O15) within 40 weeks before or 12 weeks after delivery
Unspecified hypertension (C)	Not meeting criteria for chronic or pregnancy-induced hypertension; AND Having insurance claims for diagnosis of unspecified maternal hypertension (ICD-10: O16) within 40 weeks before or 12 weeks after delivery
Preeclampsia/Eclampsia	Having insurance claims for diagnosis of preeclampsia/eclampsia (ICD-10: O14, O15) or preeclampsia superimposed on chronic hypertension (ICD-10: O11) within 40 weeks before or 12 weeks after delivery
Hypertensive disorders of pregnancy	A or B or C

ICD-10, International Classification of Diseases, 10th revision

## Results

### Magnitude of hypertension: based on the KNHNAES

The average blood pressure of Korean adults has decreased between 1998 and 2008, but there has been little change in the last 10 years. Population mean SBP/DBP level was 119/76 mmHg for Korean adults aged 20 years or older (Supplement, page 6). Over the last 20 years, the age-standardized mean blood pressure levels have decreased yet without significant change in recent years. The age-standardized prevalence of hypertension among adults aged 20 years or older also modestly decreased from 26.0% (men 29.6%, women 22.3%) in 1998 to 22.5% (men 25.9%, women 18.8%) in 2019 (Supplement, page 8). Over the same period, the age-standardized prevalence of hypertension among adults aged 30 years or older decreased from 30.8% (men 33.4%, women 27.4%) to 27.2% (men 31.1%, women 22.8%) (Supplement, page 9). However, with the rapid aging of the population, the absolute number of people with hypertension has steadily increased; as of 2019, the number has exceeded 12 million. In particular, the number of elderly women with hypertension has increased rapidly. In 2019, estimated people with hypertension was 4.34 million men and 2.78 million women under the age of 65, but 1.96 million men and 2.99 million women aged 65 years or older (Supplement, page 7).

### Hypertension management status: based on the KNHANES

In general, the hypertension management (awareness, treatment, and control rates) has improved significantly over the past two decades. In 2019, among adults aged 20 or older with hypertension, the awareness rate was 70%, the treatment rate was 63%, and the control rate was 48%. However, the degree of management varied greatly by age and sex. All management indices were better in older adults than in young adults, and higher in women than in men. However, gender-difference varies depending on age. Women under the age of 50 have higher awareness, treatment, and control rates compared to men of same age. After the age of 60, the awareness and treatment rates become similar in men and women, and the control rate is even lower in women than in men (Supplement, pages 5, 11–14).

### Healthcare utilization for hypertension: based on the NHI big data

The number of people diagnosed with hypertension has increased 3.3 times from 3 million in 2002 to 10.1 million in 2019. People receiving antihypertensive medications also increased 3.8 times from 2.5 million in 2002 to 9.5 million in 2019. More importantly, the number of people adherent to antihypertensive medication has increased more than tenfold from 0.6 million in 2002 to 6.9 million in 2019 (Supplement, page 15). The use of combination therapy has rapidly increased, with 40.6% using one class, 43.4% using two classes, and 16.0% using three or more classes of antihypertensive drug in 2018 (Supplement, page 16). In 2019, the most commonly prescribed antihypertensive drug class was ARB (72.5%), followed by CCB (60.9%), diuretics (24.7%), beta-blockers (15.7%), potassium-sparing diuretics (1.8%) and ACEi (1.8%) (Supplement, page 17). Overall, the types of antihypertensive medication were not significantly different between men and women. However, when limited to age of 20–39, monotherapy was more common in women than in men (60.0% vs. 38.1%), and ACEi/ARB prescriptions (59.5% vs. 83.7%) and CCB prescriptions were less common (48.6% vs. 61.8%) in women than in men (Supplement, page 18).

### Hypertensive disorders of pregnancy

The number of childbirths has been declining for the past 10 years, but number of pregnancies complicated with hypertension is rather increasing. In 2019, out of 298.8 thousand childbirths, 26.9 thousand women had hypertensive disorders during pregnancy. It is estimated that the prevalence of hypertensive disorder complicating pregnancies is 5.4% for chronic hypertension, 3.1% for pregnancy-induced hypertension, and 1.8% for preeclampsia/eclampsia (Supplement, page 20–21).

## Discussion

The Korea Hypertension Fact Sheet 2021 provides an overview on the magnitude and management status of hypertension in Korea. Although the population average blood pressure and the prevalence of hypertension have changed little over the last decade, the absolute number of people with hypertension have increased steadily-now exceeding 12 million due to population aging. In particular, the population of elderly women with hypertension is rapidly increasing. In addition, pregnancy-related hypertension is also increasing, requiring greater interest in preventing and managing hypertension in women.

The greatest novelty of the Korea Hypertension Fact Sheet lies on its generalizability; the KNHANES provides unbiased sampling of the Korean population, and the NHI Big Data provides medical service uses of the entire nation. However, there are some limitations to be addressed. First, the KNHANES is based on non-institutionalized residents of Korea; thus, it might not include people with severe diseases. Second, the exact collection methods and survey details varied across the KNHANES. Despite standardized protocols and rigorous quality control procedures, such variability may have affected the analysis on secular trends. Third, the NHI Big Data may not be optimal for identifying disease occurrence and prevalence, because the data have been collected for medical service claims and reimbursement purposes. Fourth, the adherence to antihypertensive medication was evaluated on a prescription basis. Thus, it is possible that adherence was overestimated, because we cannot ascertain whether the drug was actually taken or not. Fifth, we identified hypertension disorders during pregnancy based on ICD-10 code in the NHI claim database without available data on blood pressure measurements or other laboratory tests. In Korea, prenatal care is very well performed, so the detection rates of pregnancy-related disorders are presumed to be high. However, there is a possibility of misclassification in the diagnosis of hypertensive disorders during pregnancy. Finally, we were limited to anonymized datasets; therefore, the data linkage between the two datasets was unachievable.

## Conclusions

Despite the significant improvement in hypertension management over the past few decades, there is still room for further improvement. In particular, the hypertension management status was concerning in younger age groups, regardless of sex. To achieve further improvements in management, it is essential to enable early seeking for disease status and to encourage treatment in young adults. For women, prevention and management of hypertension related to pregnancy and childbirth and postmenopausal hypertension is increasingly important. To prevent complications and death from hypertension, emphasizing the importance of treatment adherence and blood pressure control remains a top priority. We also need to develop tailored prevention and management strategies that are appropriate for and inclusive of various demographics.

## Supplementary Information

**Additional file 1.**

## Data Availability

The datasets used and/or analyzed during the current study are available from the corresponding author on reasonable request.

## Abbreviations

ACEi: angiotensin-converting enzyme inhibitor
ARB: angiotensin receptor blockers
CCB: calcium channel blockers
DBP: diastolic blood pressure
ICD-10: International Classification of Diseases, 10th Revision
KNHANES: Korea National Health and Nutrition Examination Survey
NHI: National Health Insurance
NHIS: National Health Insurance Service
SBP: systolic blood pressure
