# Supplementary material for: Korea hypertension fact sheet 2021: analysis of nationwide population-based data with special focus on hypertension in women
Source: Clin Hypertens. 2022 Jan 3;28:1. doi: 10.1186/s40885-021-00188-w (PMC8722090; doi:10.1186/s40885-021-00188-w)
Supplement: Supplementary file 1 — Additional file 1. [file 40885_2021_188_MOESM1_ESM.pdf]

KOREA HYPERTENSION FACT SHEET 2021

The Korean Society of Hypertension

대한고혈압학회
THE KOREAN SOCIETY OF HYPERTENSION

Korea National Health and Nutrition Examination Survey

Subjects		Adults of age 20 years or older / 1998 to 2019
Definition of Hypertension		① Systolic blood pressure (SBP) ≥140 mmHg, or ② Diastolic blood pressure (DBP) ≥90 mmHg, or ③ Taking antihypertensive medication
Hypertension Management	Awareness rate	Proportion of people with physician diagnosis of hypertension among people with hypertension
	Treatment rate	Proportion of people taking antihypertensive medication for ≥20 days/month among people with hypertension
	Control rate (among prevalent)	Proportion of people with SBP <140 mmHg and DBP <90 mmHg among people with hypertension
	Control rate (among treated)	Proportion of people with SBP <140 mmHg and DBP <90 mmHg among people taking antihypertensive medication

Korea National Health Insurance Big Data

Subjects		Adults of age 20 years or older / 2002 to 2019
Healthcare Utilization	Diagnosis	≥1 health insurance claim for hypertension diagnosis (ICD-10: I10) each year
	Treatment	≥1 health insurance claim for hypertension diagnosis and antihypertensive prescription each year
	Adherence	Antihypertensive prescription ≥290 days (80%) each year
	Blood test	≥1 serum creatinine test each year
	Urine test	≥1 routine urinalysis or urine microalbumin test each year
Antihypertensive Treatment	Regimen	The combination of antihypertensive classes in a prescription; if the regimen is switched, one with the longest duration is selected for a given year
	Antihypertensive Class	Diuretics (DU; thiazide and related diuretics, loop diuretics), beta-blockers (BB), calcium channel blockers (CCB), angiotensin converting enzyme inhibitors (ACEi), angiotensin receptor blockers (ARB), potassium-sparing diuretics, and others (alpha-blockers, vasodilators, etc.)

Contents

4 Summary of Hypertension Statistics

6 Trends in Population Mean Blood Pressure
7 Trends in Number of People With Hypertension
8 Trends in Hypertension Prevalence
10 Trends in Hypertension Prevalence by Sex and Age

11 Trends in Awareness Rate by Sex and Age
12 Trends in Treatment Rate by Sex and Age
13 Trends in Control Rate by Sex and Age

15 Trends in Healthcare Utilization for Hypertension
16 Trends in Antihypertensive Medication Use
18 Trends in Antihypertensive Medication Use by Sex and Age
19 Trends in Complication Screening Rates by Sex and Age
20 Trends in Hypertensive Disorders of Pregnancy

Summary of Hypertension Statistics

Among Age 20+

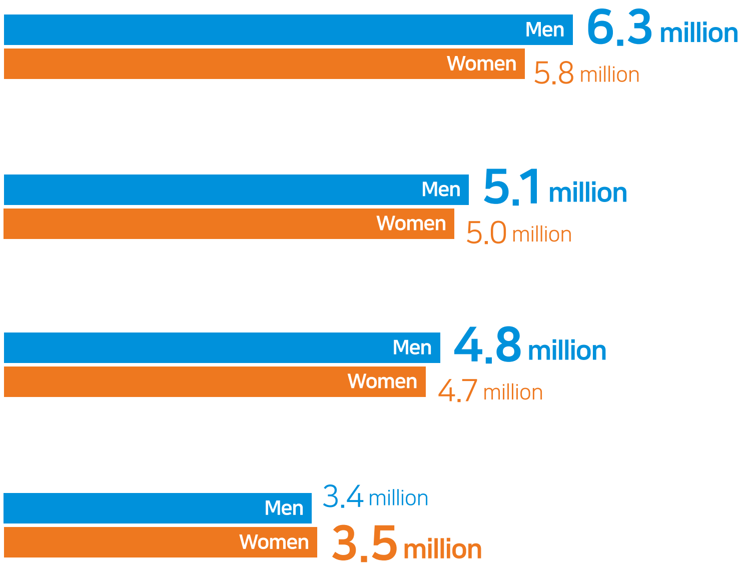

Total	
12.1 million	have hypertension
10.1 million	are diagnosed
9.5 million	are treated
6.9 million	are adherent to treatment

Among Age 65+

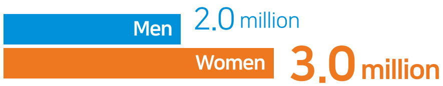

5.0 million	have hypertension

Data Source: Korea National Health and Nutrition Examination Survey 2019, Korea National Health Insurance Big Data 2019

Among Age 20+

	Overall	Men		Women
Prevalence	28%	30%	>	27%

Among Age 20+ With Hypertension

	Overall	Men		Women
Awareness Rate	70%	66%	<	75%
Treatment Rate	66%	60%	<	72%
Control Rate	48%	47%	<	49%

Among Age 65+ With Hypertension

	Overall	Men		Women
Control Rate	57%	66%	>	51%

Data Source: Korea National Health and Nutrition Examination Survey 2019

Trends in Population Mean Blood Pressure

(Age 20+, Age-Standardized*)

2019

119/76	117/76
Crude	Age-Standardized

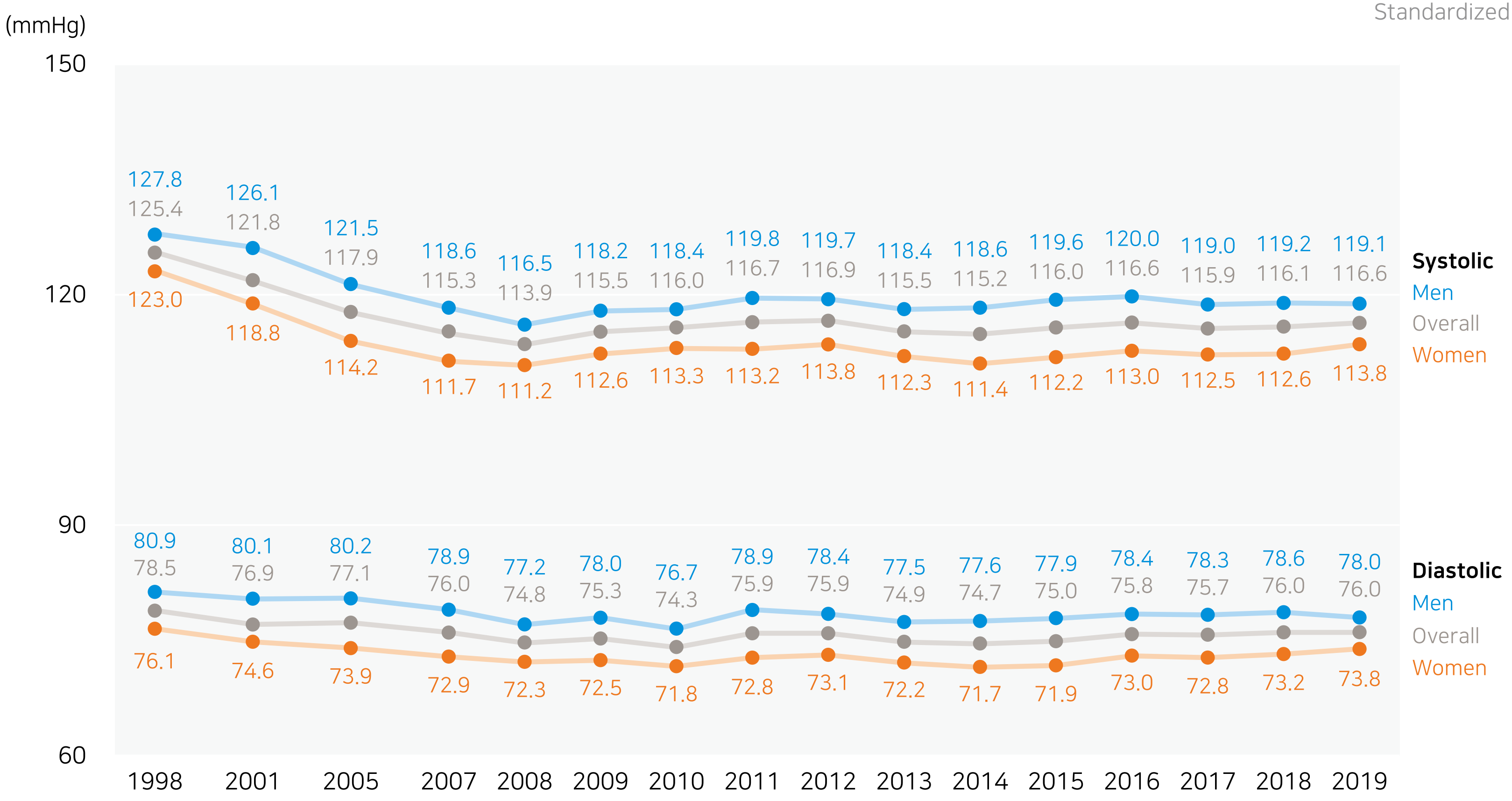

Data Source: Korea National Health and Nutrition Examination Survey 1998-2019

*Directly age-standardized to the 2005 projected population

Trends in Number of People With Hypertension

(Age 20+)

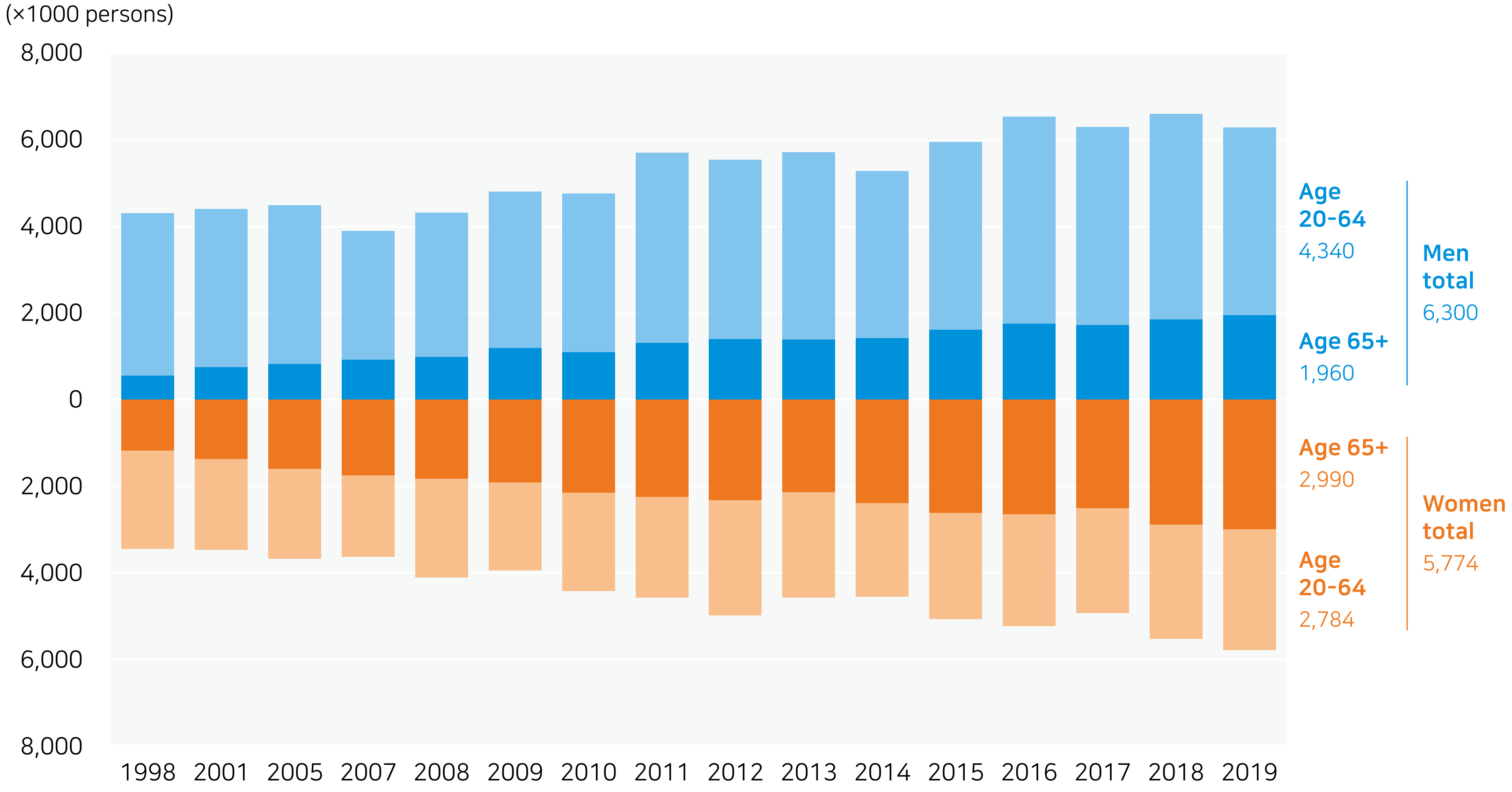

Data Source: Korea National Health and Nutrition Examination Survey 1998-2019

Trends in Hypertension Prevalence

(Age 20+, Age-Standardized*)

2019

28% Crude

23% Age-Standardized

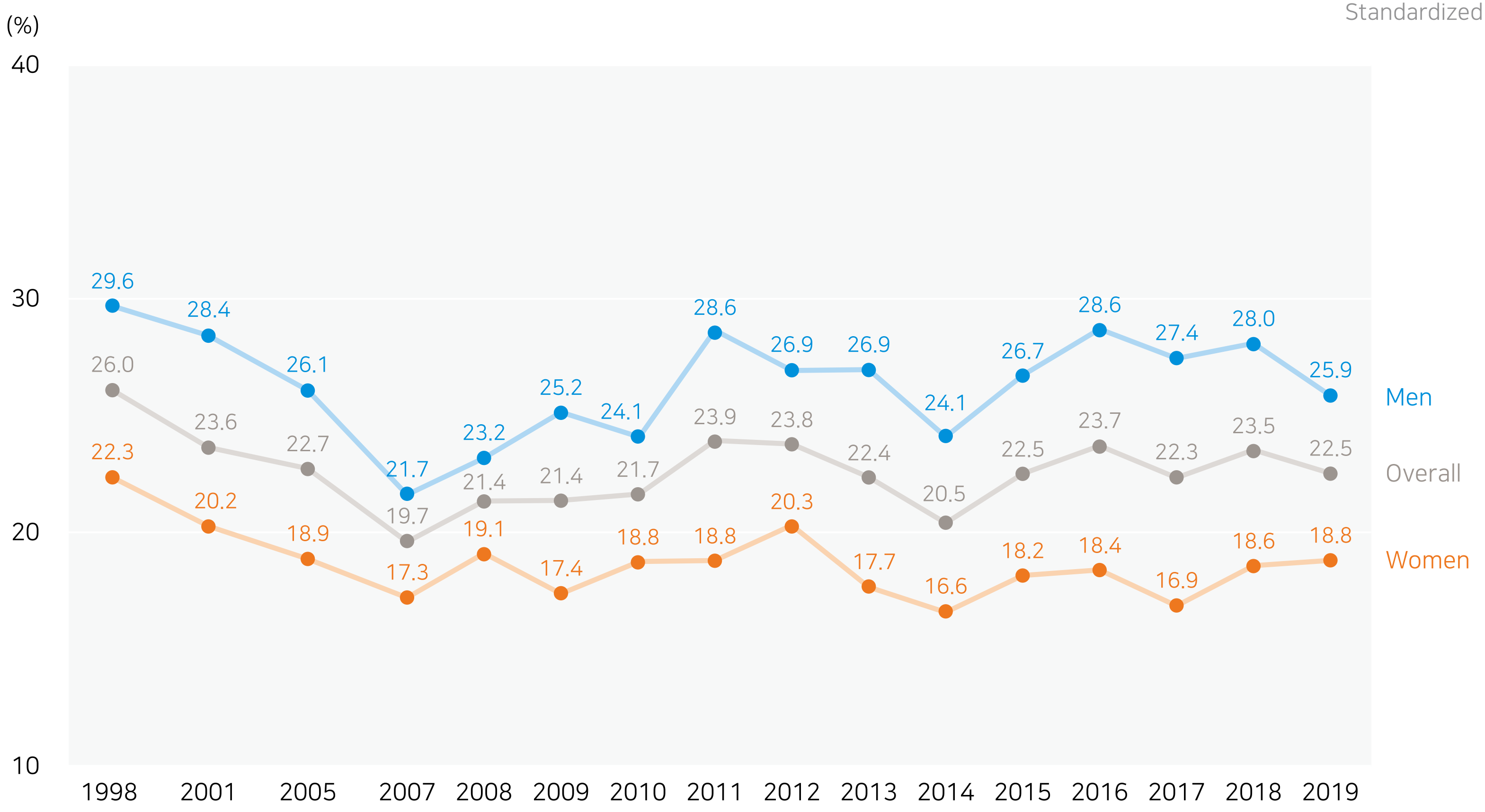

Data Source: Korea National Health and Nutrition Examination Survey 1998-2019

*Directly age-standardized to the 2005 projected population

Trends in Hypertension Prevalence

(Age 30+, Age-Standardized*)

2019

33% Crude

27% Age-Standardized

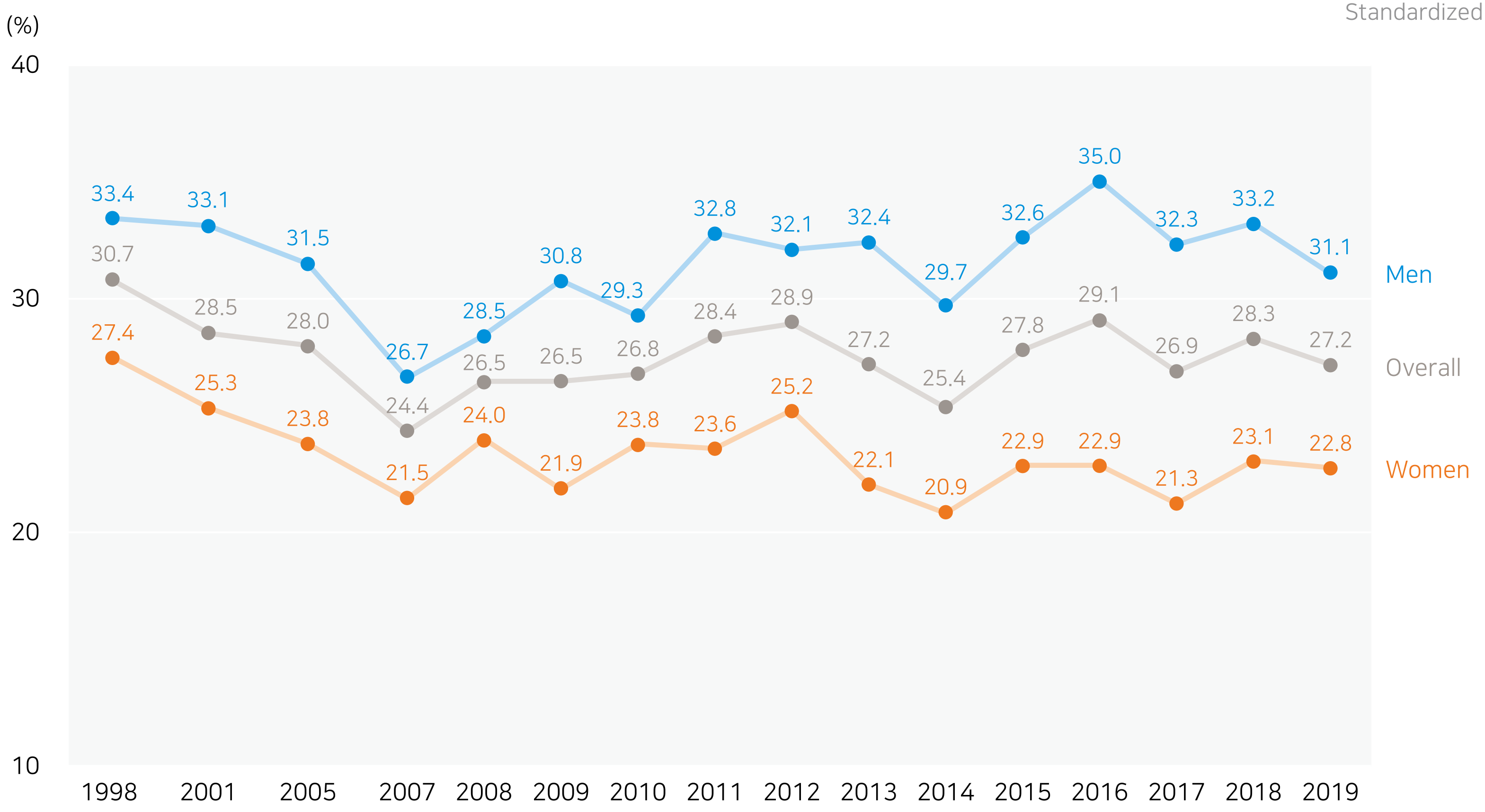

Data Source: Korea National Health and Nutrition Examination Survey 1998-2019

*Directly age-standardized to the 2005 projected population

Trends in Hypertension Prevalence

(by Sex and Age)

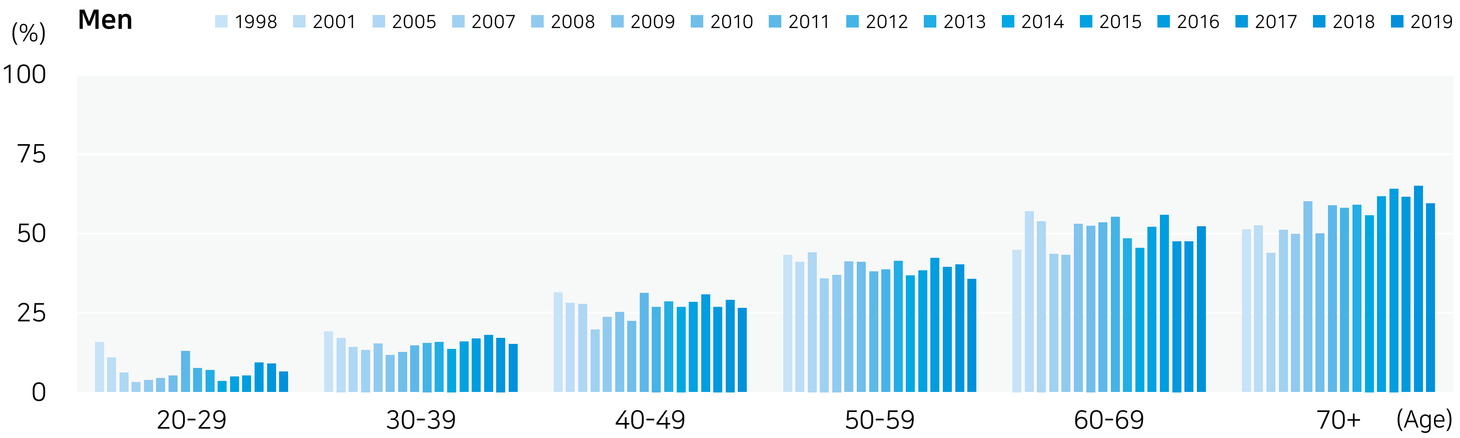

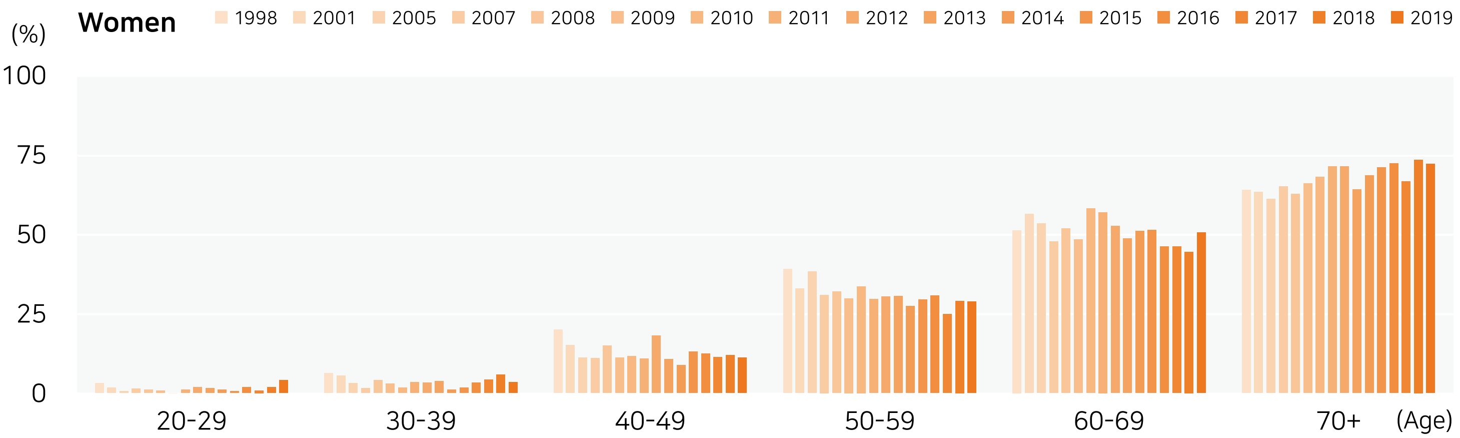

Data Source: Korea National Health and Nutrition Examination Survey 1998-2019

Trends in Awareness Rate

(by Sex and Age)

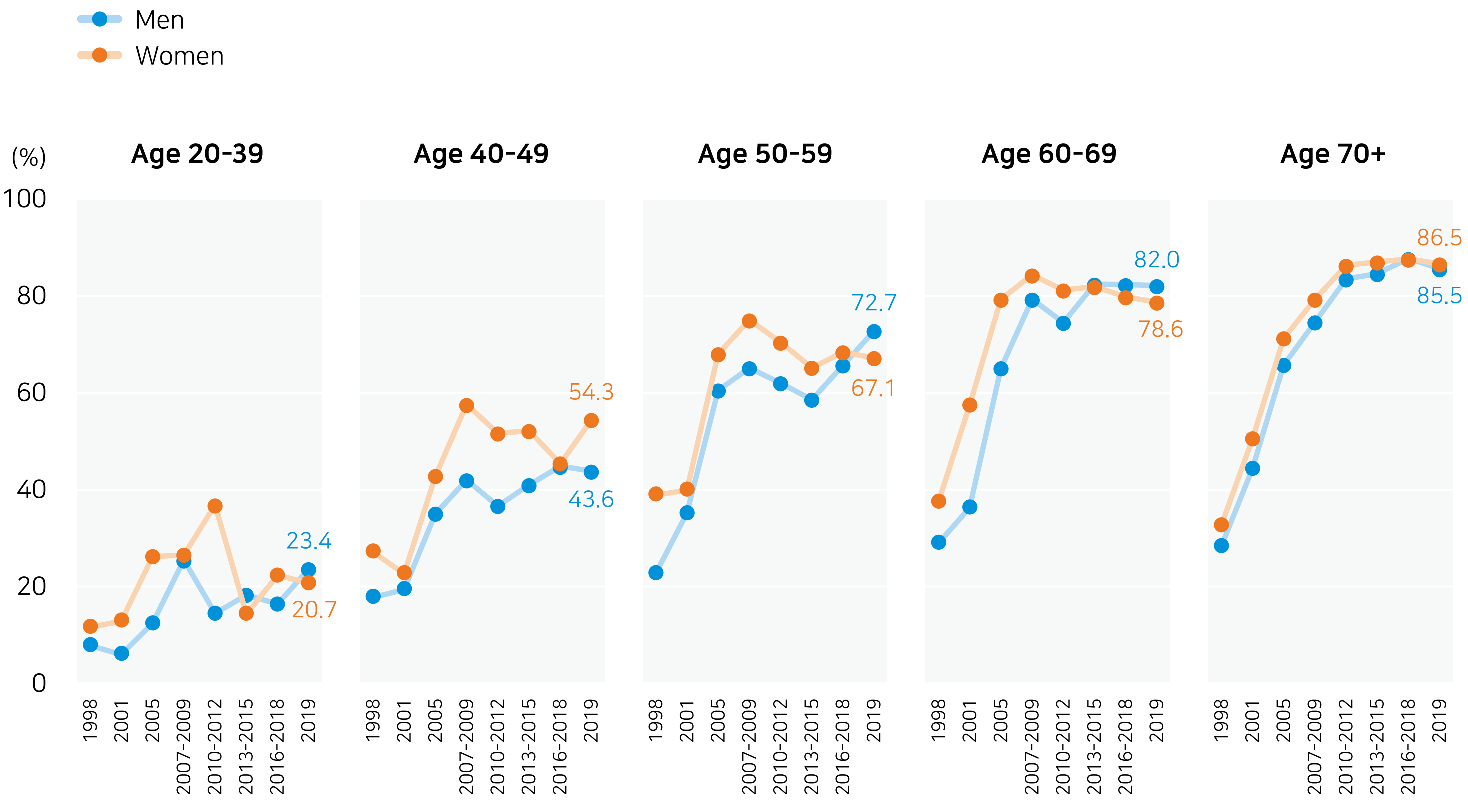

Data Source: Korea National Health and Nutrition Examination Survey 1998-2019

Trends in Treatment Rate

(by Sex and Age)

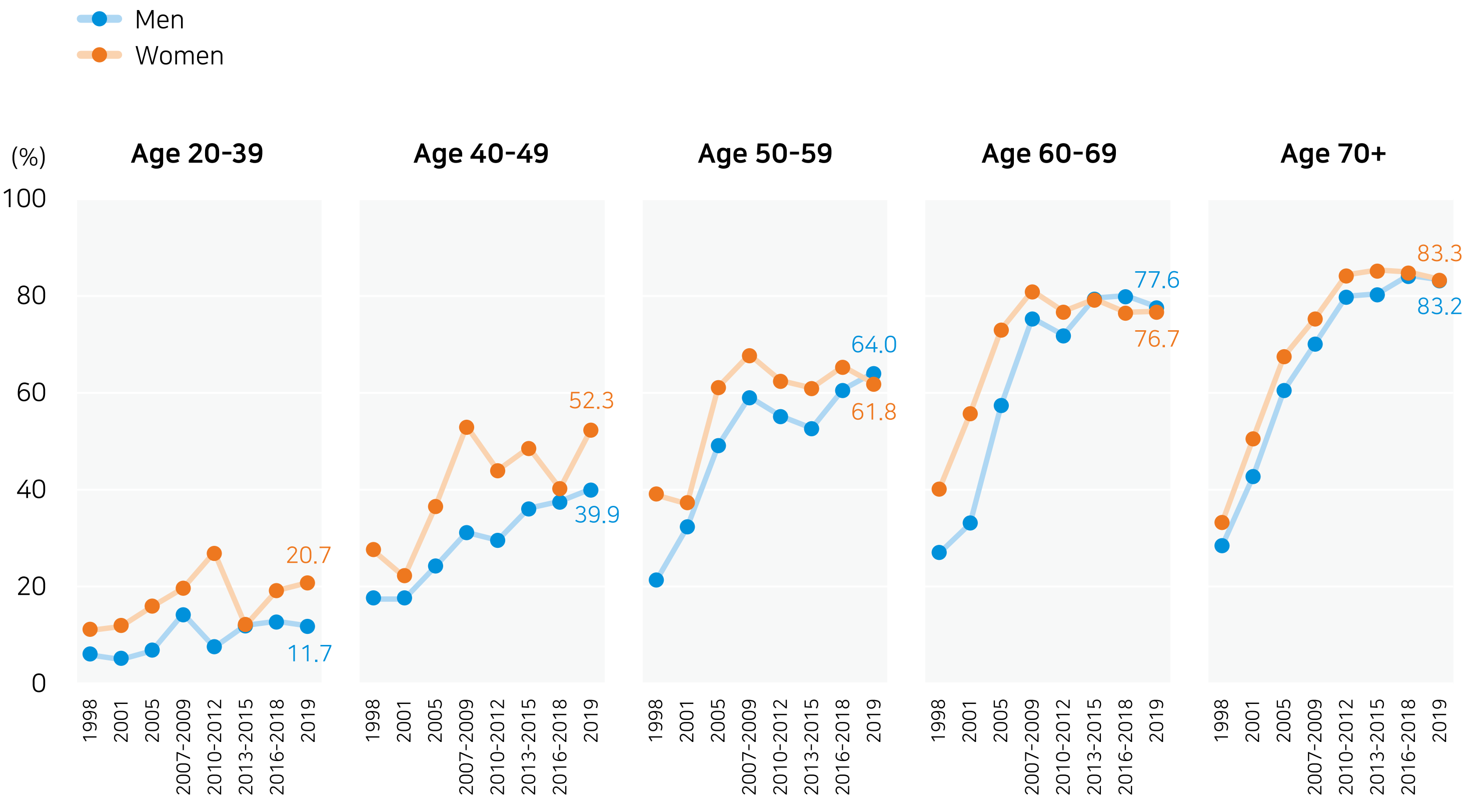

Data Source: Korea National Health and Nutrition Examination Survey 1998-2019

Trends in Control Rate (Among Prevalent)

(by Sex and Age)

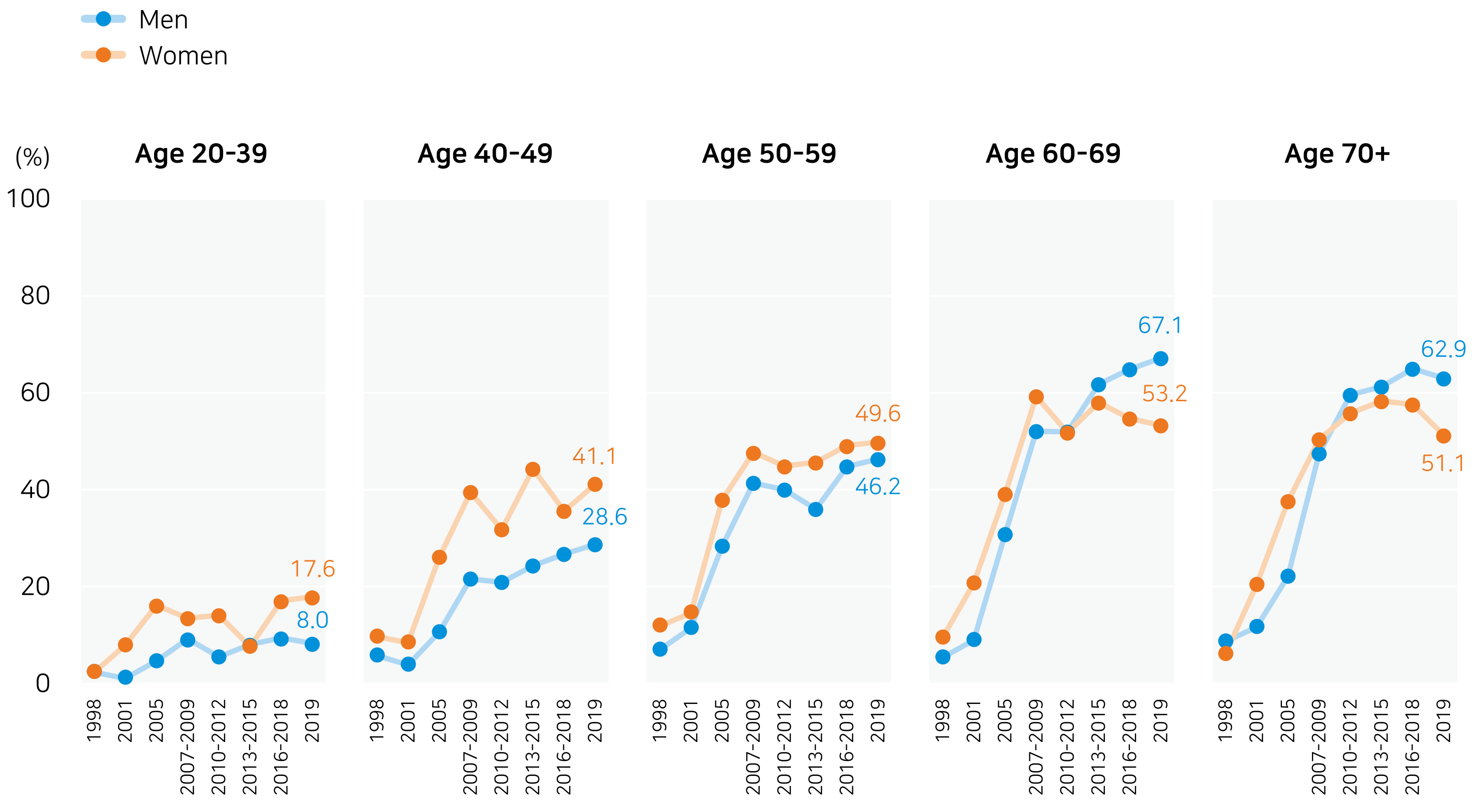

Data Source: Korea National Health and Nutrition Examination Survey 1998-2019

Trends in Control Rate (Among Treated)

(by Sex and Age)

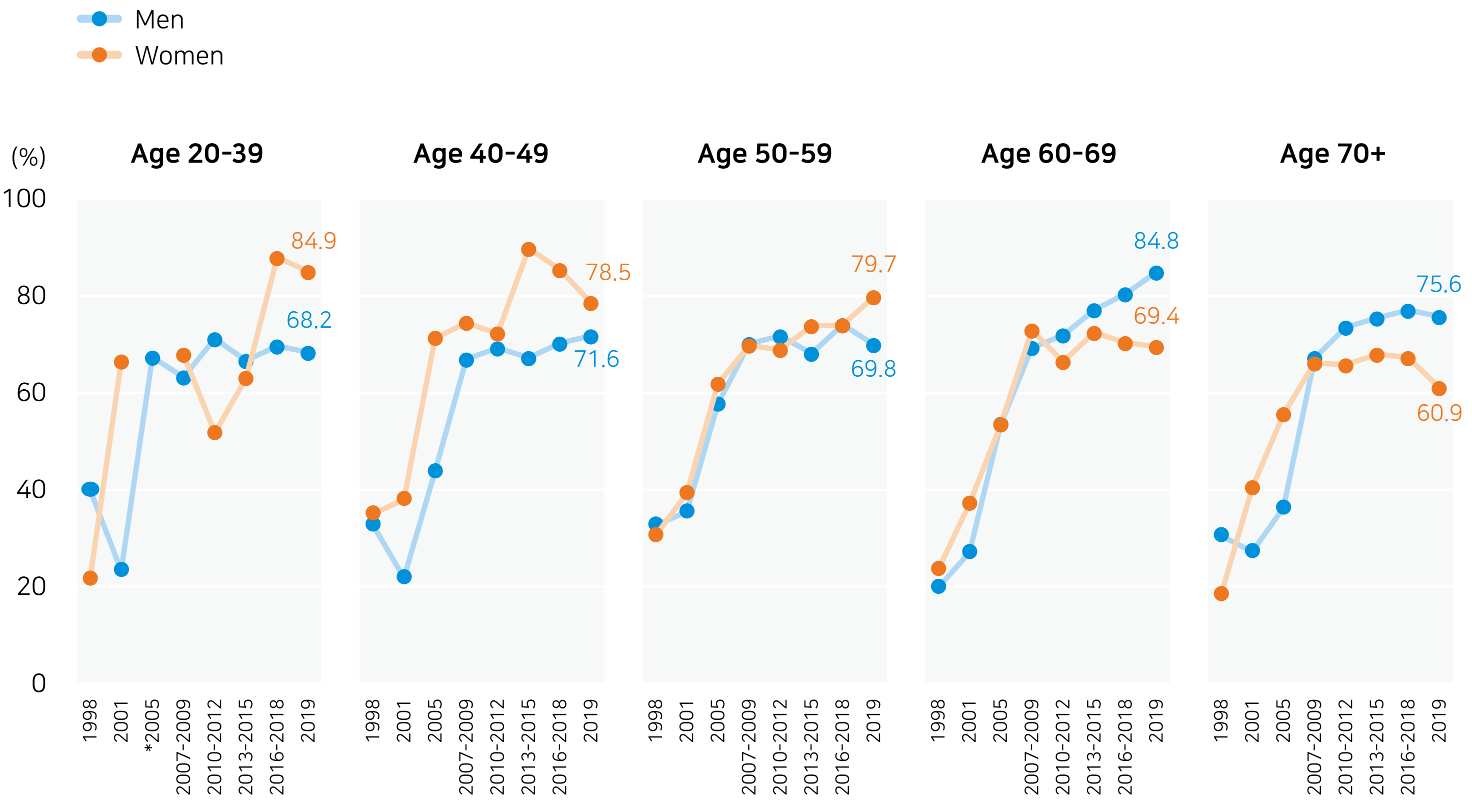

Data Source: Korea National Health and Nutrition Examination Survey 1998-2019

*Denominator <5 among women of age 20-39

Trends in Healthcare Utilization for Hypertension

(Age 20+)

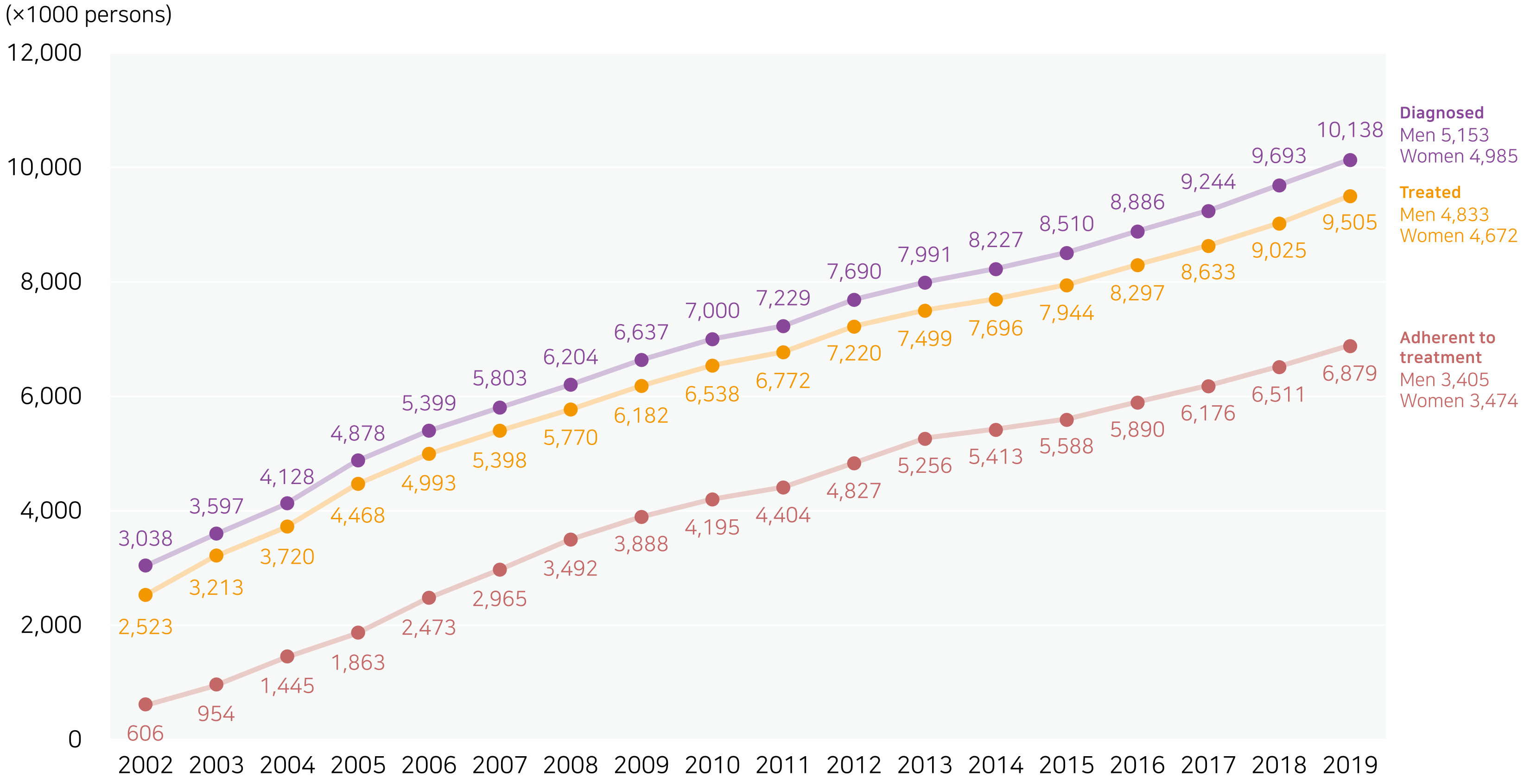

Data Source: Korea National Health Insurance Big Data 2002-2019

Trends in Antihypertensive Medication Use

(Age 20+)

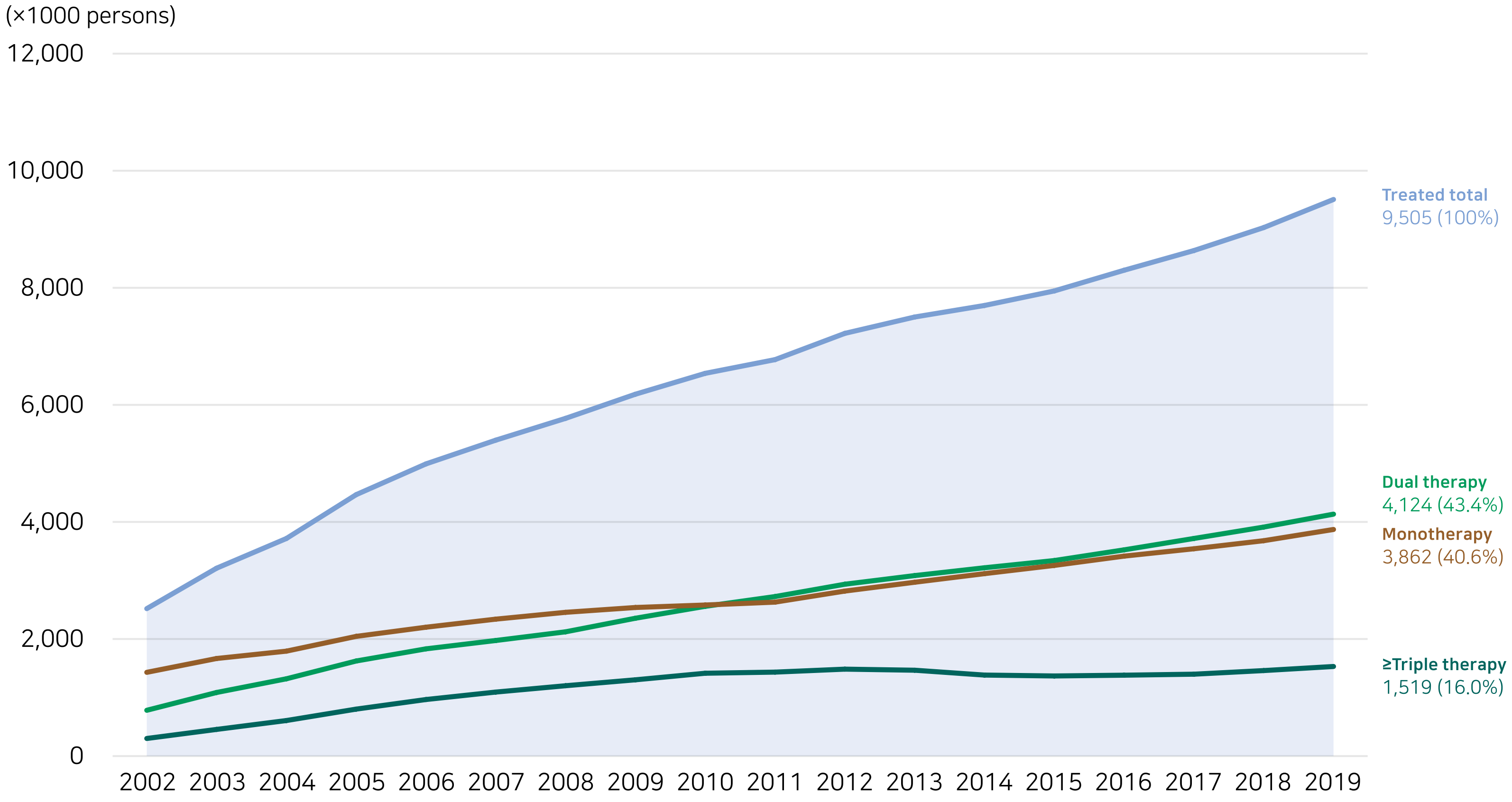

Data Source: Korea National Health Insurance Big Data 2002-2019

Trends in Antihypertensive Medication Use

(Age 20+)

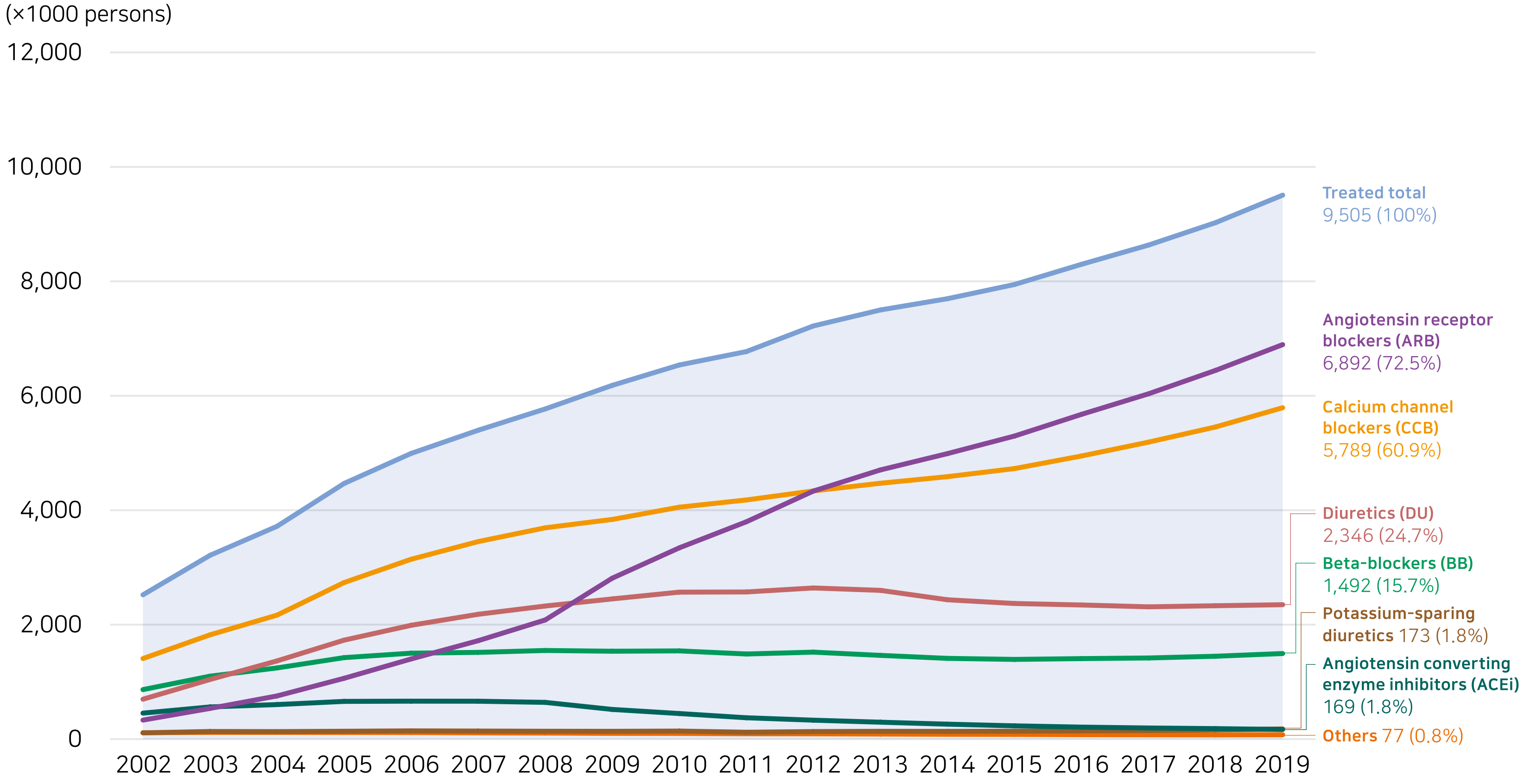

Data Source: Korea National Health Insurance Big Data 2002-2019

Trends in Antihypertensive Medication Use

(by Sex and Age)

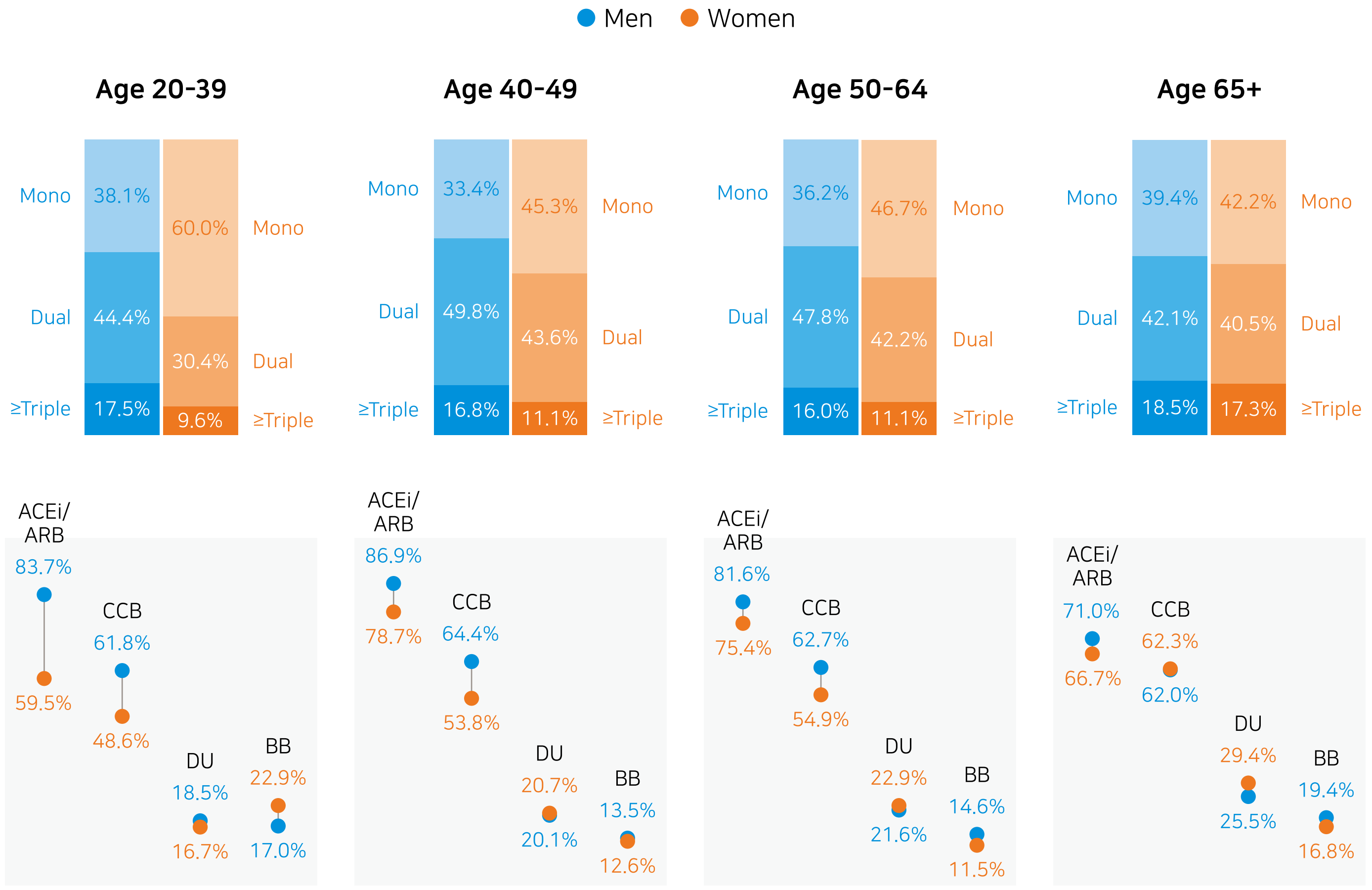

Data Source: Korea National Health Insurance Big Data 2019

Trends in Complication Screening Rates

(by Sex and Age)

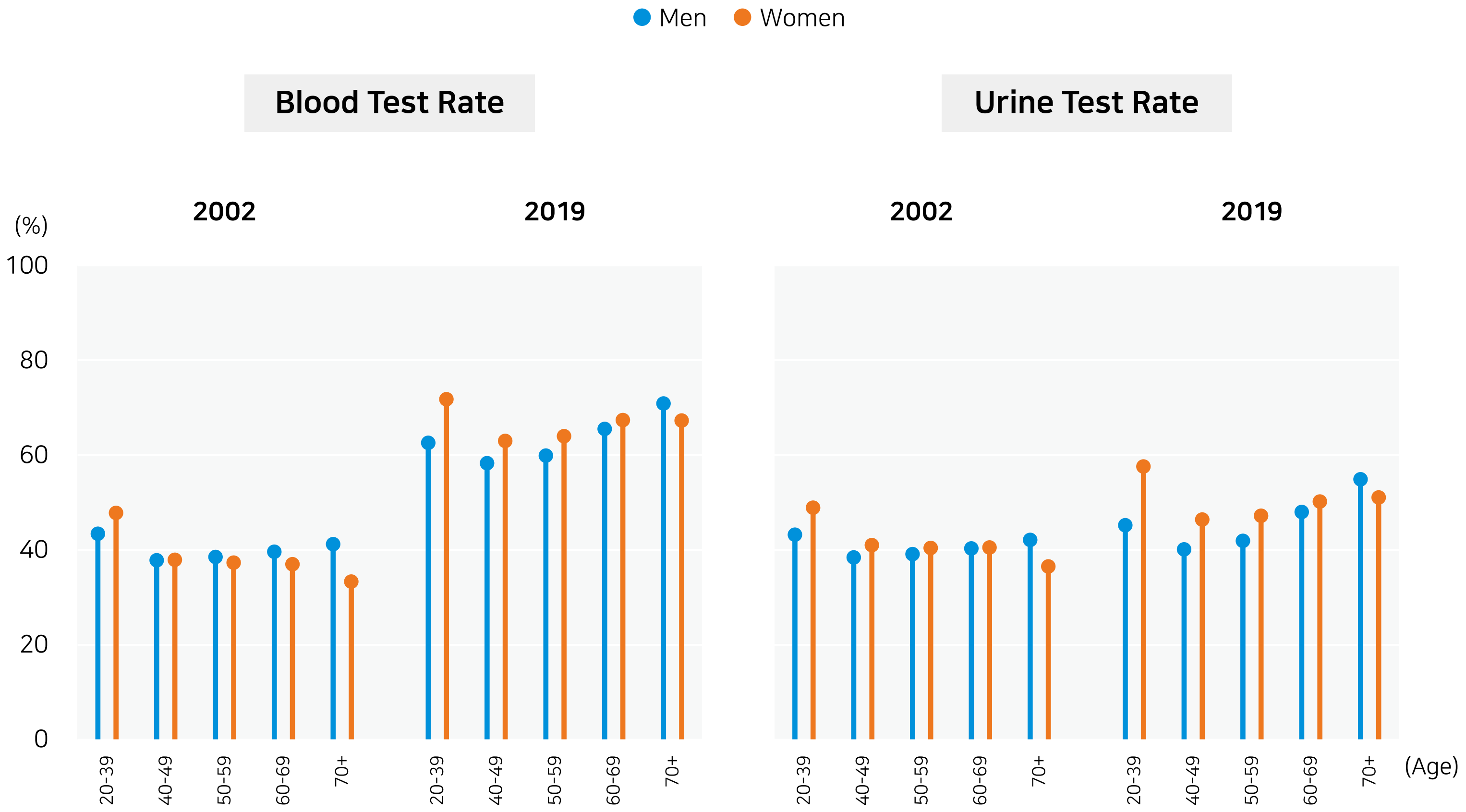

Data Source: Korea National Health Insurance Big Data 2002, 2019

Trends in Hypertensive Disorders of Pregnancy

(Age 15-49)

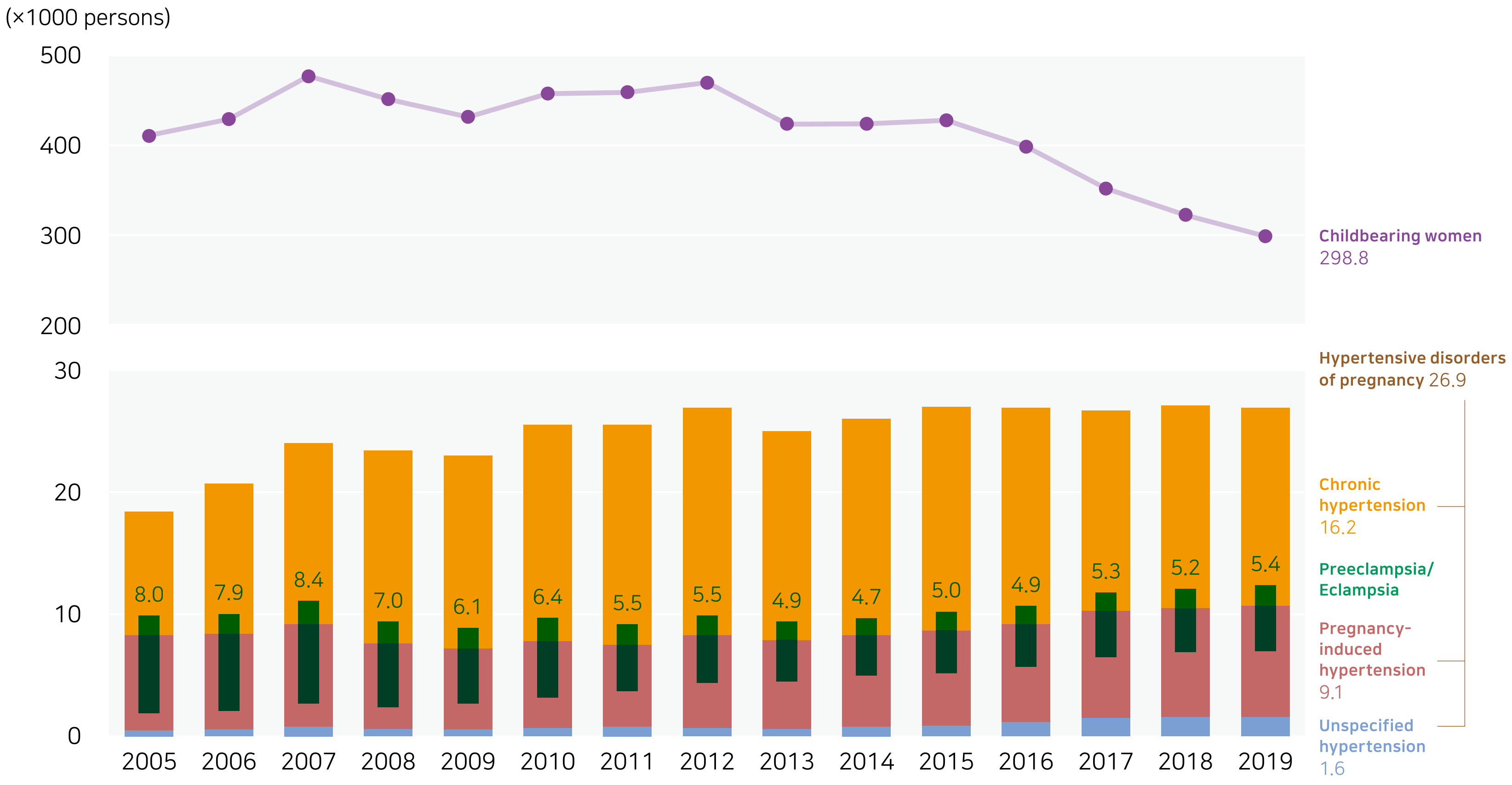

Data Source: Korea National Health Insurance Big Data 2005-2019

Trends in Hypertensive Disorders of Pregnancy

(Age 15-49, Age-Standardized* Rate Among Childbearing Women)

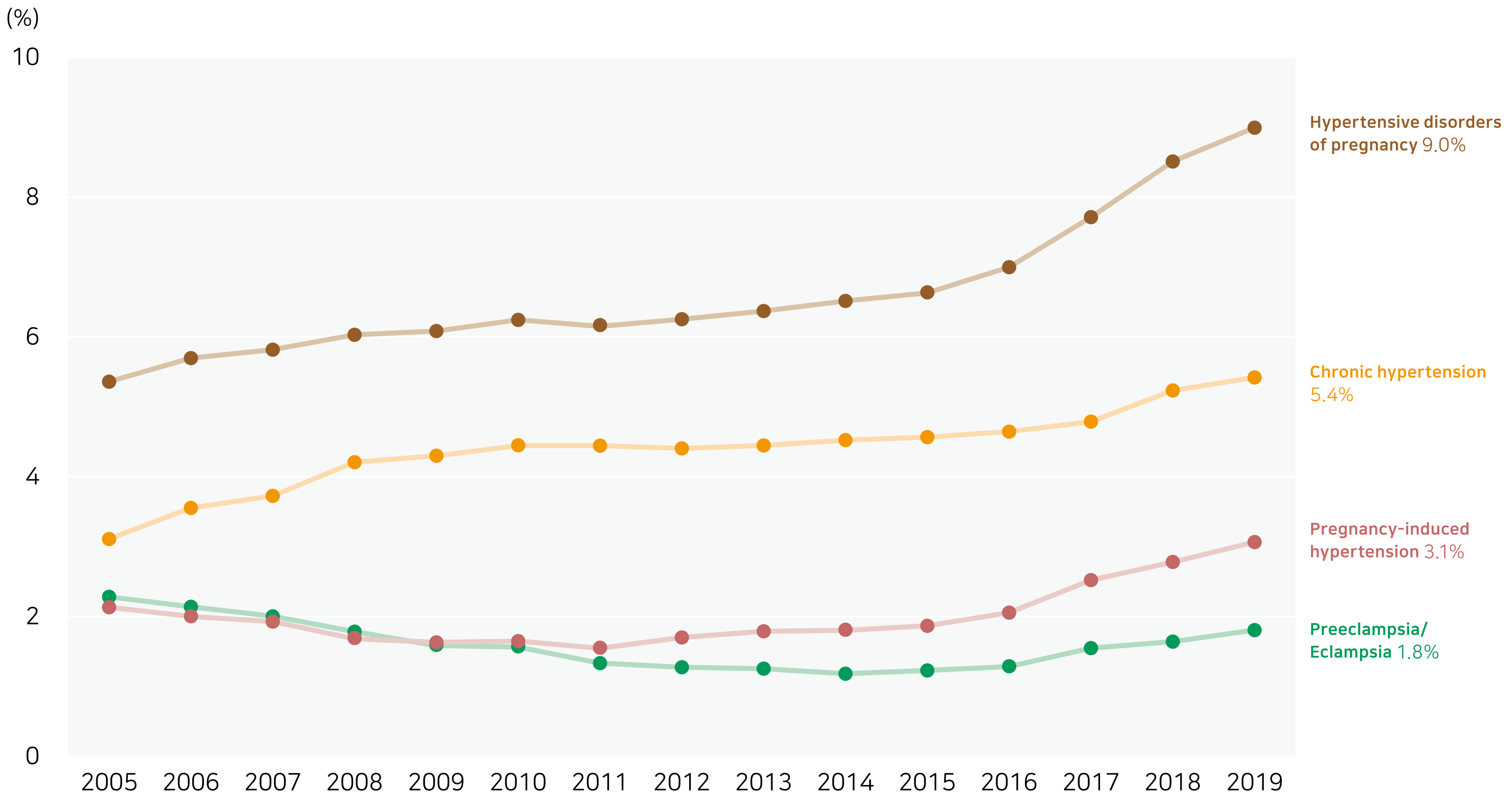

Data Source: Korea National Health Insurance Big Data 2005-2019

*Directly age-standardized to the 2019 childbearing women

KOREA HYPERTENSION FACT SHEET 2021

대한고혈압학회
THE KOREAN SOCIETY OF HYPERTENSION

This fact sheet will be published in *Clinical Hypertension*,
the official journal of the Korean Society of Hypertension

www.koreanhypertension.org
